# Phenotypic and Genetic Diversity of *Hydrangea* Germplasm Resources and the Construction of DNA Fingerprint Profiles

**DOI:** 10.3390/plants15182821

**Published:** 2026-09-14

**Authors:** Tongfen Xu, Ao Li, Mei Zhou, Yanxia Jia, Zhenyi Yang, Zenghua Yang, Yandi Wu, Chao Wang

**Affiliations:** 1 Yunnan Key Laboratory of Landscape Plant Resource Cultivation and Application, Yunnan Province Engineering Research Center for Functional Flower Resources and Industrialization, Key Laboratory of Forest Disaster Warning and Control in Yunnan Province, Southwest Forestry University, Kunming 650224, China; tf3368007177@163.com (T.X.); 18993707390@163.com (A.L.); 18362754439@163.com (M.Z.); wuyandi@swfu.edu.cn (Y.W.); 2 Kunming Institute of Botany, Chinese Academy of Sciences, Kunming 650201, China; jiayanxia@mail.kib.ac.cn; 3 Yunnan Yuyong Hydrangea Innovation Technology Co., Ltd., Kunming 652501, China; diesworder@163.com (Z.Y.); 17783947790@163.com (Z.Y.)

**Keywords:** *Hydrangea*, morphological traits, SSR markers, genetic diversity, fingerprint profiles

## Abstract

This study assessed the genetic diversity of 30 *Hydrangea accessions* (21 wild species and 9 cultivated varieties) using 33 morphological traits and 22 pairs of polymorphic SSR markers. Morphological analysis showed that for 24 qualitative traits, the Shannon–Wiener index (H′) ranged from 0.124 to 1.559, the Simpson index (D) ranged from 0.260 to 0.816, and the Pielou evenness index (E) ranged from 0.179 to 0.948; for the 9 quantitative traits, H′ ranged from 0.534 to 1.267, E ranged from 0.486 to 0.972, and the coefficient of variation ranged from 11.53% to 70.76%, This indicates that phenotypic variation in quantitative traits is rich and evenly distributed. Principal component analysis identified 9 principal components with a cumulative contribution of 76.998%; comprehensive membership function evaluation was used to screen for germplasm with excellent overall traits, such as *H. m.* ‘Hua kai fu gui’, *H. m.* ‘Bo da lan’, and *H. m.* ‘Jin se shi yun’. SSR analysis amplified a total of 30 alleles, with the number of effective alleles (Ne) ranging from 3.838 to 23.041, the Shannon diversity index (I) ranging from 1.647 to 3.362, and the polymorphism information content (PIC) ranging from 0.710 to 0.979 (mean 0.894). The Mantel test revealed no significant correlation between morphological and molecular data (r = 0.003, *p* = 0.517), indicating that the two types of data provide complementary information. Cluster analysis based on morphological traits and SSR markers classified the tested germplasm into 4 and 2 clusters, respectively, both of which effectively distinguished wild species from cultivated varieties; the PCA results were consistent with the clustering results. As few as three pairs of SSR markers were sufficient to distinguish all 30 accessions; using eight pairs of core primers, DNA fingerprint profiles and corresponding QR codes were successfully constructed for each accession. The results indicate that combining morphological traits with SSR markers is an effective approach for the identification, conservation, and breeding utilization of *Hydrangea* germplasm resources.

## 1. Introduction

*Hydrangea* (*Hydrangea* macrophylla (Thunb.) Ser.) is native to China and is a traditional Chinese flower. It belongs to the order Cornales, family Hydrangeaceae, and genus *Hydrangea* L. It has also been known by other names, such as Ziyanghua, Baxianhua, and Fentuanhua. The genus *Hydrangea* comprises approximately 73 species worldwide, with 47 species and 11 varieties distributed in China, representing a very rich genetic resource [1,2]. Southwestern China is one of the important centers of diversity for this genus. *Hydrangeas* feature large inflorescences, diverse flower forms, a wide variety of colors, and a long flowering period. As an important ornamental plant in landscaping, they are highly adaptable to cultivation and are widely used for garden beautification, potted ornamental displays, and cut-flower production, possessing extremely high economic and landscape value [3,4].

*Hydrangeas* have a long history of cultivation, broad ecological adaptability, and rich genetic resources, with significant diversity in flower and leaf traits—although these morphological traits are easily influenced by genetic factors and environmental conditions. They provide a more comprehensive and intuitive illustration of the basic characteristics of genetic resources [5,6,7]. Morphological traits form the basis for the identification and classification of germplasm resources, providing an intuitive and comprehensive reflection of their fundamental characteristics. *Hydrangeas* exhibit rich diversity in traits such as inflorescence morphology (subspherical, flat-disk, and conical), the number of sepals, variations in flower color (whether or not they exhibit blue coloration), leaf shape, and plant habit exhibit rich diversity [8]. Authoritative taxonomic works such as the *Flora of China* have established a morphological classification system for *Hydrangea* species based on inflorescence type, leaf shape, and trichome characteristics, laying the foundation for the systematic classification of the genus [9,10]. A systematic survey of *Hydrangea* resources in Hunan Province revealed that the province is a center of distribution for the genus. A comparative study of stomatal and epidermal trichome characteristics further revealed morphological differences among different species [11]. Morel et al. conducted a systematic analysis of the morphological structure of the subspecies *H. aspera* and found that morphological analysis based on axial structure and growth units can effectively distinguish germplasm with significant genetic differences [12]. However, morphological traits are easily influenced by environmental conditions, cultivation practices, and growth and development stages; moreover, morphological differences between some varieties are subtle. Relying solely on morphological markers makes it difficult to accurately distinguish among closely related germplasm, thereby limiting the precise identification and effective utilization of *Hydrangea* germplasm resources.

Molecular marker technology does not rely on morphological traits or external environmental conditions; it can directly reveal genetic variation and phylogenetic relationships among accessions at the DNA level, thereby overcoming the limitations of traditional morphological analysis. Among these, SSR (Simple Sequence Repeat) markers, also known as microsatellite DNA, are widely used in plant genetic diversity analysis, germplasm identification, and fingerprinting due to their high polymorphism, high information content, experimental stability, strong reproducibility, and codominant inheritance [13]. SSR markers can accurately identify genetic differences among germplasm and rapidly identify varieties, providing a reliable molecular basis for the conservation and utilization of germplasm resources.

SSR markers have already been applied to a certain extent in *Hydrangea* species. Rinehart et al. [14] used 38 SSR markers to construct a phylogenetic tree of *Hydrangea* germplasm resources and corrected the nomenclature of some *Hydrangea* species. Hempel et al. [15] established the Wädenswil pedigree and corrected the names of individual *Hydrangeas* within it. Yamamoto et al. [16] conducted a genetic structure study on 188 accessions using SSR molecular markers, with 19 accessions of *Hydrangea* serrata collected in Japan and 14 accessions collected on Jeju Island, South Korea. Ito et al. [17] conducted an SSR molecular marker study on four yellow-veined *Hydrangea* accessions collected from South Korea and Japan, identifying five SSR loci with distinct bands from 23 pairs of primers. Based on these five SSR markers, Choi et al. [18] conducted a comparative analysis of Korean yellow-veined *Hydrangeas* and three Japanese yellow-veined *Hydrangea* Cultivated varieties. The results showed that two multi-locus genotypes present in the 285 Korean individuals were absent from the Japanese samples, leading to the hypothesis that Korean yellow-veined *Hydrangeas* may have originated from Japanese introductions. Additionally, Waki et al. [19] used 93 F2-generation *Hydrangea* plants derived from a cross between ‘Kirakiraboshi’ and ‘Frauyoshimi’ as material. Employing next-generation sequencing technology, they developed 672 SSR markers and constructed the first genetic linkage map for *Hydrangea*. Wu et al. [20] established an F1 population by crossing the *Hydrangea* Cultivated varieties ‘Veitichii’ and ‘Endless Summer.’ They then integrated 267 SSR polymorphic markers developed from the transcriptome and 3923 SNP markers obtained via GBS technology—totaling 4190 polymorphic loci—to construct paternal, maternal, and integrated genetic linkage maps, respectively. Although the aforementioned studies have provided a large number of SSR markers for analyzing the genetic diversity of the *Hydrangea* genus, most of them were developed based on Japanese or European Cultivated varieties. Preliminary experiments indicate that the amplification success rate of these published primers in native Chinese *Hydrangea* germplasm is generally low (less than 30%), and the proportion of polymorphic loci is limited, making it difficult to meet the needs of this study for a detailed evaluation of Chinese germplasm resources. Currently, a comprehensive fingerprinting system for *Hydrangea* germplasm resources has not yet been established. The SSR markers for *Hydrangea* reported in the existing literature were published some time ago, and the primers selected are unlikely to meet the current needs for constructing genetic maps of *Hydrangea* germplasm. Furthermore, existing studies have largely focused on the application of single molecular markers, and research combining morphological traits with SSR molecular markers for a comprehensive evaluation of *Hydrangea* germplasm resources remains rare.

In recent years, significant progress has been made in research on the molecular systematics and genetic regulation of key traits in *Hydrangea* species. At the molecular systematics level, phylogenetic analyses based on chloroplast genome sequences have provided new molecular evidence for species delineation and subgenus classification within the genus *Hydrangea* [21]. With regard to the genetic mechanisms underlying flower colour, the key regulatory genes for *Hydrangea* flower colour have been progressively elucidated, providing a molecular basis for targeted breeding for flower colour [22]. Furthermore, the breeding of heat-tolerant varieties has achieved phased results, laying the foundation for the promotion and application of hydrangeas in the humid and hot regions of southern China [23]. However, whilst these studies have largely focused on molecular systematics, the genetic regulation of specific traits, or variety breeding techniques, the systematic evaluation of genetic diversity and the construction of DNA fingerprint profiles for China’s indigenous *Hydrangea* germplasm resources remain relatively underdeveloped.

Although the aforementioned studies have provided important references for the molecular systematics, genetic regulation of flower colour, and variety breeding of the *Hydrangea* genus, the application of the integrated use of morphological traits and molecular markers in the comprehensive evaluation of *Hydrangea* germplasm resources remains relatively limited. Morphological traits are influenced by the environment and growth and development stages, whereas molecular markers cannot directly reflect differences in morphological traits. Combining these two methods can provide complementary insights, enabling a more comprehensive and accurate assessment of genetic diversity within germplasm [24,25]. In recent years, scholars both domestically and internationally have made significant progress in the evaluation of *Hydrangea* germplasm resources. Qu Lianwei et al. [26] conducted research on artificial hybridization techniques for *Hydrangeas*; Hai Feng et al. [27] conducted breeding work on new *Hydrangea* varieties. However, these studies have largely focused on cultivation techniques and variety breeding, lacking systematic analysis of the genetic background and phylogenetic relationships of germplasm resources. Although the genus *Hydrangea* exhibits high species diversity globally, systematic evaluations of genetic diversity and the construction of DNA fingerprint profiles for native Chinese *Hydrangea* germplasm resources remain relatively underdeveloped. In this study, using 30 *Hydrangea* accessions (including 21 wild species and 9 cultivated varieties) as materials, we conducted genetic diversity analysis based on morphological trait surveys. We utilized a total of 22 SSR primer pairs—comprising 16 newly developed pairs and 6 pairs reported in the literature—to calculate genetic parameters such as the number of alleles (Na), effective number of alleles (Ne), Shannon diversity index (I), observed heterozygosity (Ho), expected heterozygosity (He), and polymorphism information content (PIC). We analyzed genetic similarity among the accessions and established an SSR fingerprint profile. The consistency between morphological and molecular data was assessed using the Mantel test. A comprehensive analysis of the genetic diversity and phylogenetic relationships of *Hydrangea* germplasm was conducted to provide a theoretical basis and technical support for the identification, classification, conservation, and molecular marker-assisted breeding of *Hydrangea* germplasm resources.

## 2. Results

### 2.1. Analysis of Phenotypic Diversity in Hydrangea Germplasm

#### 2.1.1. Differences in Plant Morphological Characteristics

**Figure 1:**
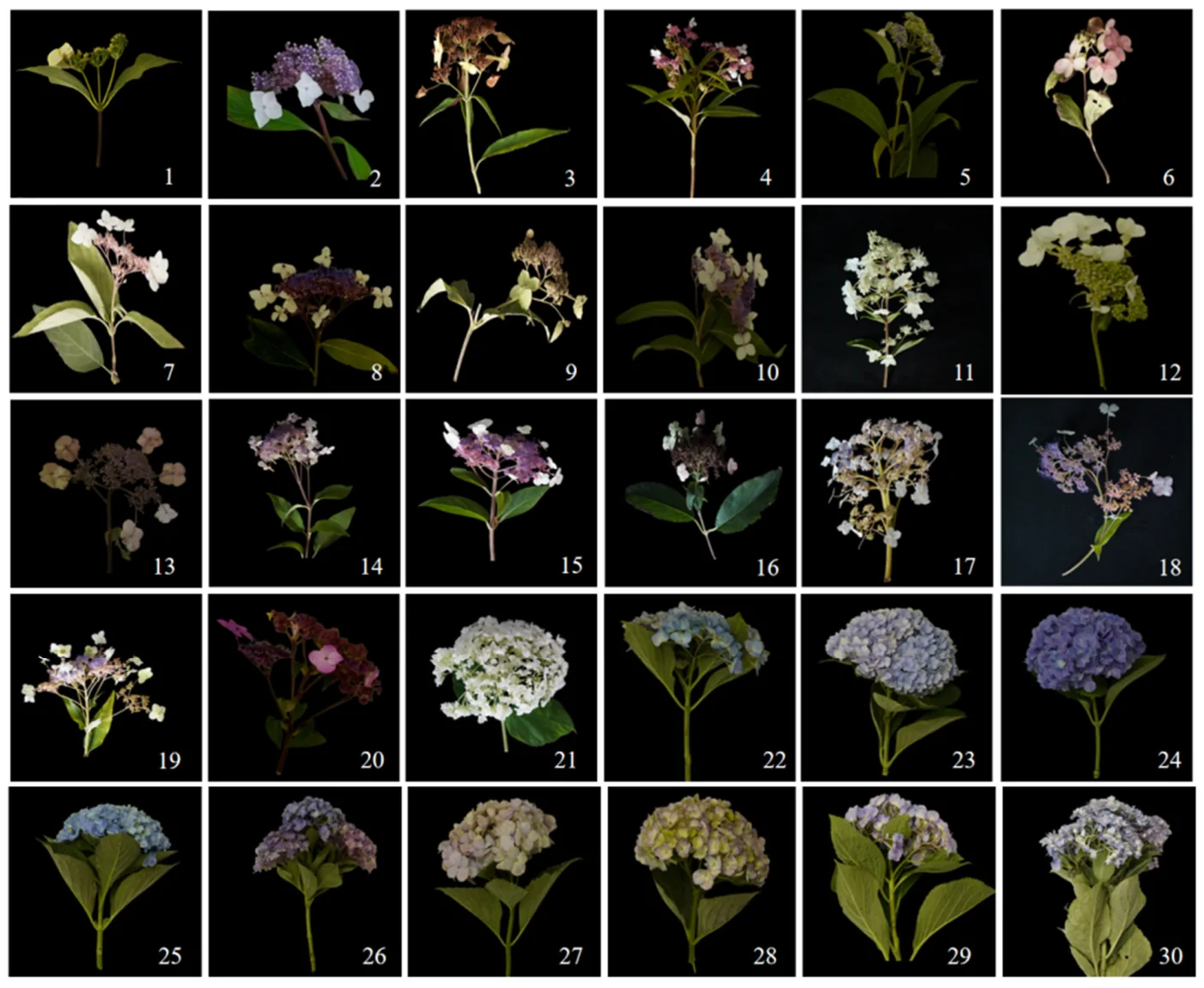
Floral organ characteristics of 30 *Hydrangea* accessions. Note: 1. *H.chinensis.* Maxim; 2. *H. davidii* Franch; 3. *H. linkweiensis* Chun; 4. *H. vinicolor* Chun; 5. *H. stenophylla* Merr. & Chun; 6. *H. gracilis* W. T. Wang & M. X. Nie; 7. *H. coenobialis* Chun; 8. *H.kwangtungensis* Merr.; 9. *H. caudatifolia* W. T. Wang & M. X. Nie; 10. *H. zhewanensis* P. S. Hsu & X. P. Zhang; 11. *H. paniculata* Siebold; 12. *H. hypoglauca* Rehder; 13. *H. macrocarpa* Hand.-Mazz; 14. *H.strigosa* Rehder; 15. *H. aspera*; 16. *H. longipes* Franch; 17. *H. robusta* Hook. f. & Thomson; 18. *H. kawakamii* Hayata; 19. *H. longiflolia* Hayata; 20. *H. coacta* C. F. Wei; 21. *H. anomala* D. Don; 22. *H. m.* ‘Bo da lan’; 23. *H. m.* ‘Ma mei li’; 24. *H. m.* ‘Qiao ban lan’; 25. *H. m.* ‘Fan hua’; 26. *H. m.* ‘Lan tian’; 27. *H. m.* ‘Ren mian tao hua’; 28. *H. m.* ‘Jin se shi yun’; 29. *H. m.* ‘Hua kai fu gui’; 30. *H. m.* ‘Xing kong’; Numbers 1 to 21 represent wild species, whilst numbers 22 to 30 represent cultivated varieties. For each accession, both the overall morphology of the inflorescence and a close-up of a single sterile flower are shown.

**Figure 2:**
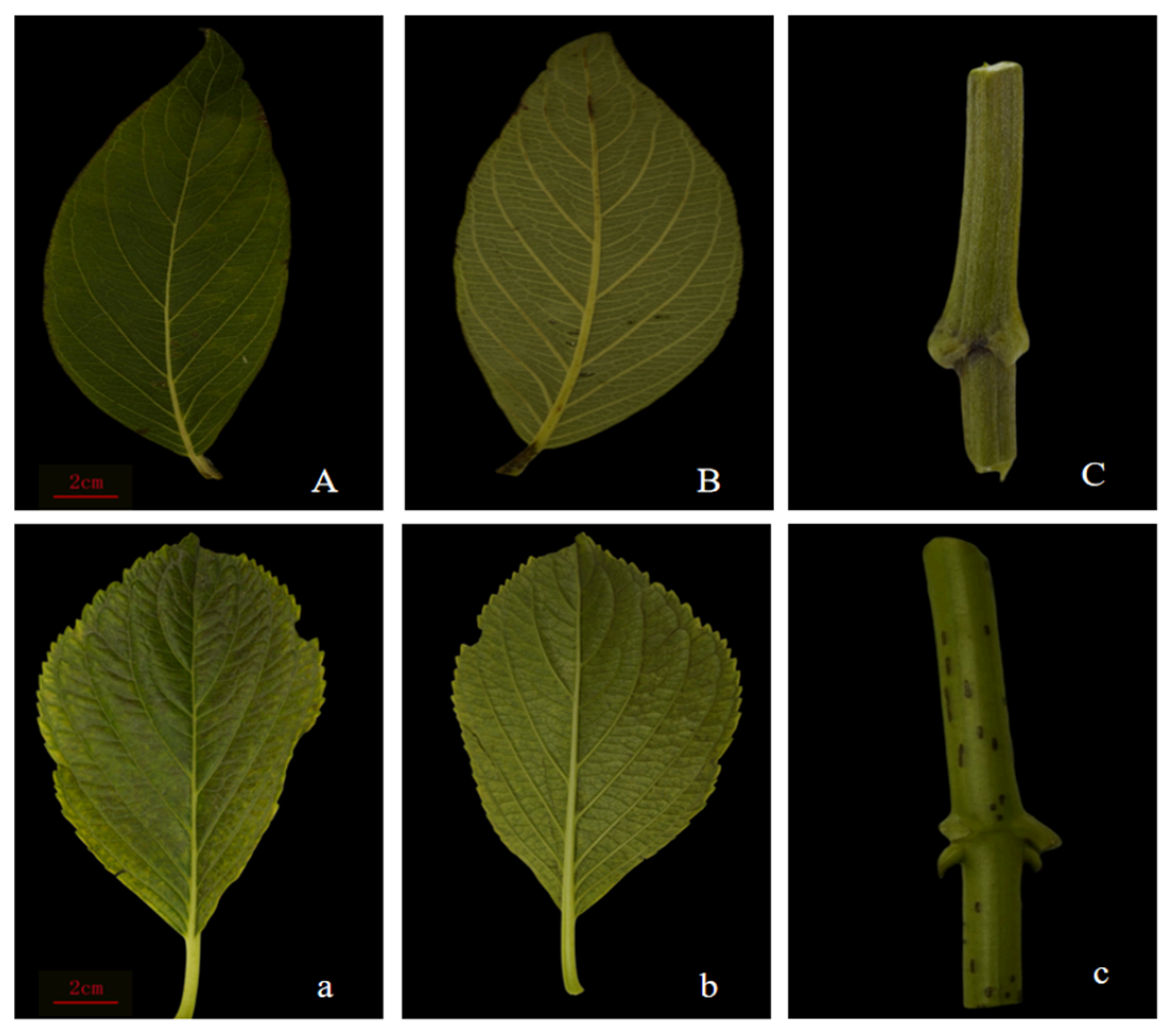
Morphological changes in wild and cultivated varieties. Note: (**A**–**C**) represent wild varieties, and (**a**–**c**) represent cultivated varieties.

The 21 *Hydrangea* wild-type accessions and 9 cultivated varieties analyzed exhibited rich morphological diversity in floral, leaf, and stem characteristics (Figure 1). Regarding floral characteristics, flower colors encompassed six types: white, green, pink, yellow, blue, and purple. Among these, wild species were predominantly white and pink, while cultivated varieties exhibited a wider variety of blue and purple variations; inflorescence shapes included four types: flattened, flattened-globose, globose, and subconical. Wild species were mostly flattened (with clearly visible fertile flowers), whereas cultivated varieties had evolved into flattened-globose or globose forms (with dense clusters of sterile flowers); the apical shape of sterile calyx segments is classified into two types—rounded-blunt and pointed—while the margin notching exhibits three states: no notching, partial notching, and entire margins; the dominant color of sterile calyx segments is particularly diverse in cultivated varieties (Figure 1). Regarding leaf characteristics, leaf shapes encompass six types: ovate, narrow-elliptic, elliptic, broadly elliptic, round, and obovate. The depth of leaf margin serrations exhibits a continuous gradient ranging from absent or extremely weak to extremely strong. Hairiness on the leaf underside is common in wild types (86.7%), whereas cultivated varieties are all glabrous. The glossiness of the upper leaf surface is “strong” in all cultivated varieties, whereas it is mostly “absent or weak” in the wild type, reflecting the improvement in leaf texture through artificial selection (Figure 2). Regarding stem characteristics, stem color includes three types: green, brown, and black, with black observed only in (*H. gracilis* W. T. Wang & M. X. Nie); the size and number of stem lenticels were mostly “small” and “absent or very few” in wild species, whereas cultivated varieties exhibited a gradient of variation ranging from “medium” to “few” to “many”; stem pubescence was widespread in wild species (93.3%), while all cultivated varieties were glabrous (Figure 2). In summary, morphological variation in *Hydrangea* germplasm exhibits a distribution pattern characterized by diverse floral traits, a gradient in vegetative organs, and intensive cultivation in cultivated varieties, providing an important basis for germplasm identification and directed breeding.

#### 2.1.2. Analysis of Phenotypic Diversity in Quality Traits

Twenty-four quality traits were evaluated across 30 wild and cultivated *Hydrangea* accessions. Analysis of genetic diversity for these quality traits (as shown in Table 1) indicated varying degrees of Phenotypic diversity among the tested *Hydrangea* accessions. The Shannon–Weaver diversity index (H′) ranged from 0.124 to 1.559 (mean 0.721), with the highest value observed for the primary color of sterile sepals (SCSP) (H′ = 1.559), indicating rich variation in floral ornamental traits, while the lowest value was observed for the inward-curving margin of sterile sepals (SSIC) (H′ = 0.124), reflecting a highly conserved trait. The Simpson index (D) and Pielou evenness index (E) averaged 0.581 and 0.670, respectively. Among trait categories, floral traits (particularly those related to sterile calyx segments) exhibited the highest diversity, with SCSP (H′ = 1.559) and inflorescence shape (IM, H′ = 1.123) showing the most significant variation; stem traits exhibited moderate diversity (H′ = 0.637–0.995); among leaf traits, leaf shape (BS, H′ = 1.362) and dentate margin depth (DSL, H′ = 1.137) showed the highest diversity. Notably, leaf upper surface gloss (LUSG) and leaf lower surface pubescence (AP) had the highest Pielou evenness (both 0.948), indicating uniform distribution; whereas SSIC (E = 0.179), the number of whorls of sterile sepals (NWSC, E = 0.353), and the number of folds on sterile sepals (SSWF, E = 0.354) had the lowest uniformity, with frequency distributions highly concentrated in the dominant class (>86%). Floral traits, particularly the morphology of sterile sepals, are the primary sources of phenotypic variation in the tested *Hydrangea* germplasm and can provide foundational data for the selection of ornamental traits and the conservation of germplasm resources.

#### 2.1.3. Analysis of Phenotypic in Quantitative Traits

Analysis of the coefficient of variation (CV), Shannon–Weaver diversity index (H′), and Pielou evenness index (E) for nine quantitative traits in wild and cultivated *Hydrangea* varieties indicates that *Hydrangea* germplasm exhibits rich phenotypic variation in continuous traits (as shown in Table 2). Since the degree of dispersion for quantitative traits is already reflected by CV and range, and H′ comprehensively incorporates information on richness and evenness, the Simpson’s index (D) is not recalculated here to avoid redundancy. The CV ranges for each trait were 11.53% to 70.76%, with an average of 36.32%. Among these, petiole length (PL) had the highest CV (70.76%) and the greatest genetic variation; the number of sepals per flower (NSPL) had the lowest CV (11.53%), indicating the greatest stability and strong genetic conservatism. In terms of range, leaf length (LGL) exhibited the greatest variation (18.20 cm), while the number of sepals per flower (NSPL) showed the smallest variation (2.00), consistent with the CV results. The Shannon–Weaver diversity index (H′) ranged from 0.534 to 1.267, with a mean of 1.092; plant height (PH) and the diameter of a single sepal (DSS) had the highest H′ values (both 1.267), while the number of sepals per flower (NSPL) had the lowest (0.534). The Eranged from 0.486 to 0.972, with a mean of 0.821; the E value was highest for the logarithm of lateral leaf veins (NLV) (0.972) and lowest for the number of sepals per flower (NSPL) (0.486), indicating that the distribution of the latter was extremely uneven across the different grades. In summary, leaf traits (such as leaf length and width) and floral organ size traits (such as the diameter, length, and width of a single sepal) are the primary sources of quantitative trait variation in the tested germplasm and possess high potential for genetic improvement; they should be prioritized as key traits in *Hydrangea* germplasm evaluation and the selection of breeding parents; in contrast, the number of sepals per flower is highly conserved and carries relatively limited weight in variety identification.

### 2.2. Comprehensive Evaluation of Morphological Traits in Hydrangea Germplasm

#### 2.2.1. Principal Components of Morphological Traits

The raw dataset consisted of 33 morphological traits from wild and cultivated *Hydrangea* germplasm. Qualitative traits were encoded as micro-dummy variables, while quantitative traits were standardized using Z-scores, and the data were merged. Subsequent analysis via principal component analysis (PCA) (as shown in Table 3) indicated that the eigenvalues of the first nine principal components were all greater than 1.5, with a cumulative contribution rate of 76.998%, which essentially reflected most of the information contained in the original traits. Among these, PC1 had the highest contribution rate (23.951%), with negative loadings primarily from petiole color (PC, −0.893) and sterile sepal margin notching (SSNM, −0.812), and positive loadings from sterile sepal overlap degree (ODSS, 0.939), sterile calyx lobe density (SSD, 0.800), petiole length (PL, 0.733), and leaf upper surface gloss (LUSG, 0.668). PC1 can be defined as the “composite factor of vegetative organs and inflorescence morphology,” reflecting the synergistic variation among leaf morphology, stem characteristics, and the arrangement of sterile floral organs in *Hydrangea* germplasm. PC2 contributed 9.886%, with major negative loadings from leaf dominant color (LDC, −0.657) and leaf margin serration depth (DSL, −0.646), and positive loadings from number of stem lenticels (NLS, −0.543) and leaf shape (BS, −0.449), though the absolute values were low. PC2 primarily represents the “leaf color and leaf margin morphology factor,” revealing a genetic association between leaf pigment deposition and leaf margin structure. PC3 contributed 8.544%, with major negative loadings from plant height (PH, −0.572) and the cross-sectional shape of sterile sepals (CSSS, −0.372), while positive loadings came from stem lenticel size (SS, 0.685) and the number of sepals per flower (NSPF, 0.553), reflecting a positive correlation between stem structural traits and floral numerical traits. It is worth noting that the first three principal components collectively explained 42.381% of the total variation. Among these, floral traits (particularly the morphology and density of sterile sepals) and leaf morphology were the primary drivers of phenotypic differentiation in *Hydrangea* germplasm. These findings provide a theoretical foundation for subsequent phenotype-based germplasm clustering and the selection of breeding parents.

**Figure 3:**
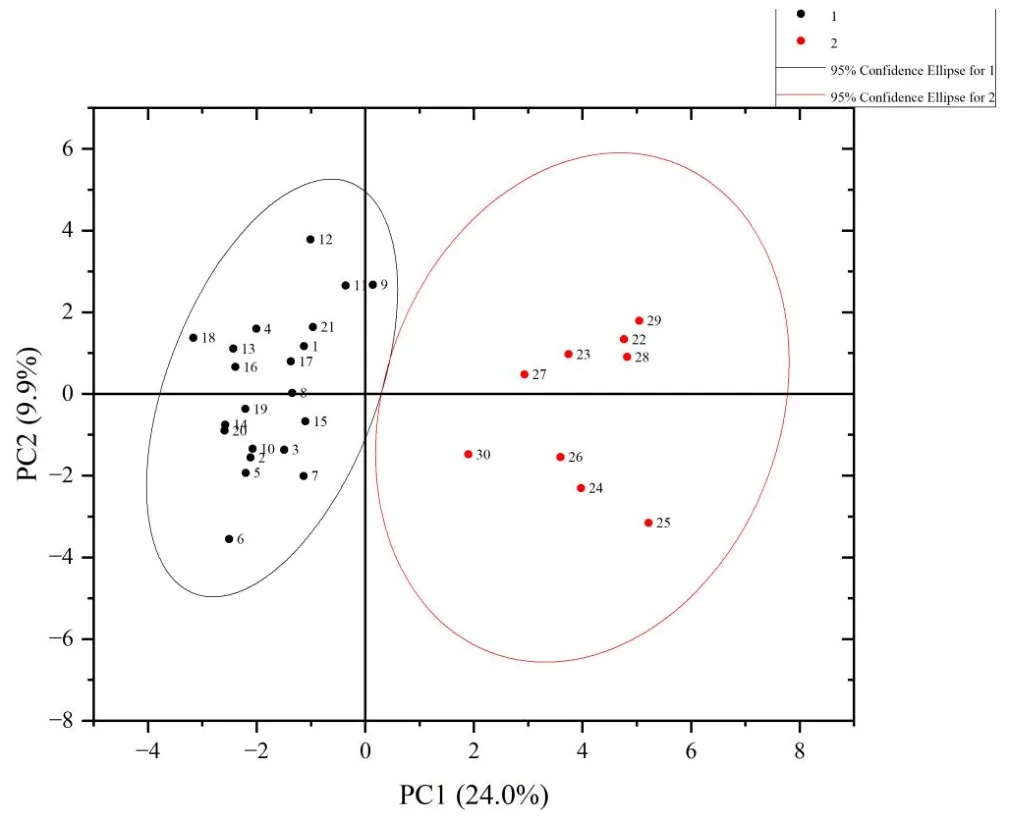
Scatter plot of the principal component analysis (PCA) of 30 *Hydrangea* accessions based on morphological traits.

To provide a more intuitive illustration of the distribution pattern of the test germplasm in the principal component space, a principal component scatter plot was plotted using PC1 and PC2 as the coordinate axes (Figure 3). As shown in the figure, the 30 hydrangea accessions exhibit a distinct pattern of differentiation in the principal component space. Along the PC1 axis, Accession 1 (*H. chinensis*), Accession 2 (*H. davidii*) and Accession 3 (*H. linkweiensis*) are distributed on the positive half-axis (PC1 > 4.0), with scores significantly higher than those of the other accessions. This indicates that these three wild species possess a unique combination of traits under the ‘composite factor of vegetative organs and inflorescence morphology’, primarily reflected in the distinctive expression of traits such as petiole colour, notches on the margins of sterile sepals, and the degree of overlap of sterile sepals; the PC1 scores of the remaining 27 accessions ranged from −4.0 to 0.0, showing no obvious cluster differentiation along the PC1 axis. Along the PC2 axis, all nine cultivated varieties (accessions 22–30) clustered in the high-score region of the positive half-axis of PC2 (PC2 ranging from 22.0 to 30.0), whilst the PC2 scores of the 21 wild accessions were widely distributed across the range of 1.0 to 25.0. This indicates that cultivated varieties exhibit a highly consistent combination of traits along the ‘leaf colour and margin morphology factor’ (dark green dominant leaf colour and shallow serration along the leaf margin), whereas the dispersed distribution of wild accessions along this axis reflects the rich natural variation they retain in traits such as leaf colour and serration. Overall, cultivated varieties cluster in a specific region within the principal component space, forming a clear separation from most wild types along the PC2 axis. This indicates that artificial selection has caused cultivated varieties to converge towards a specific combination pattern in terms of leaf traits, whilst also further validating the reliability of principal component analysis for morphological traits.

#### 2.2.2. Correlation Analysis of Morphological Traits

**Figure 4:**
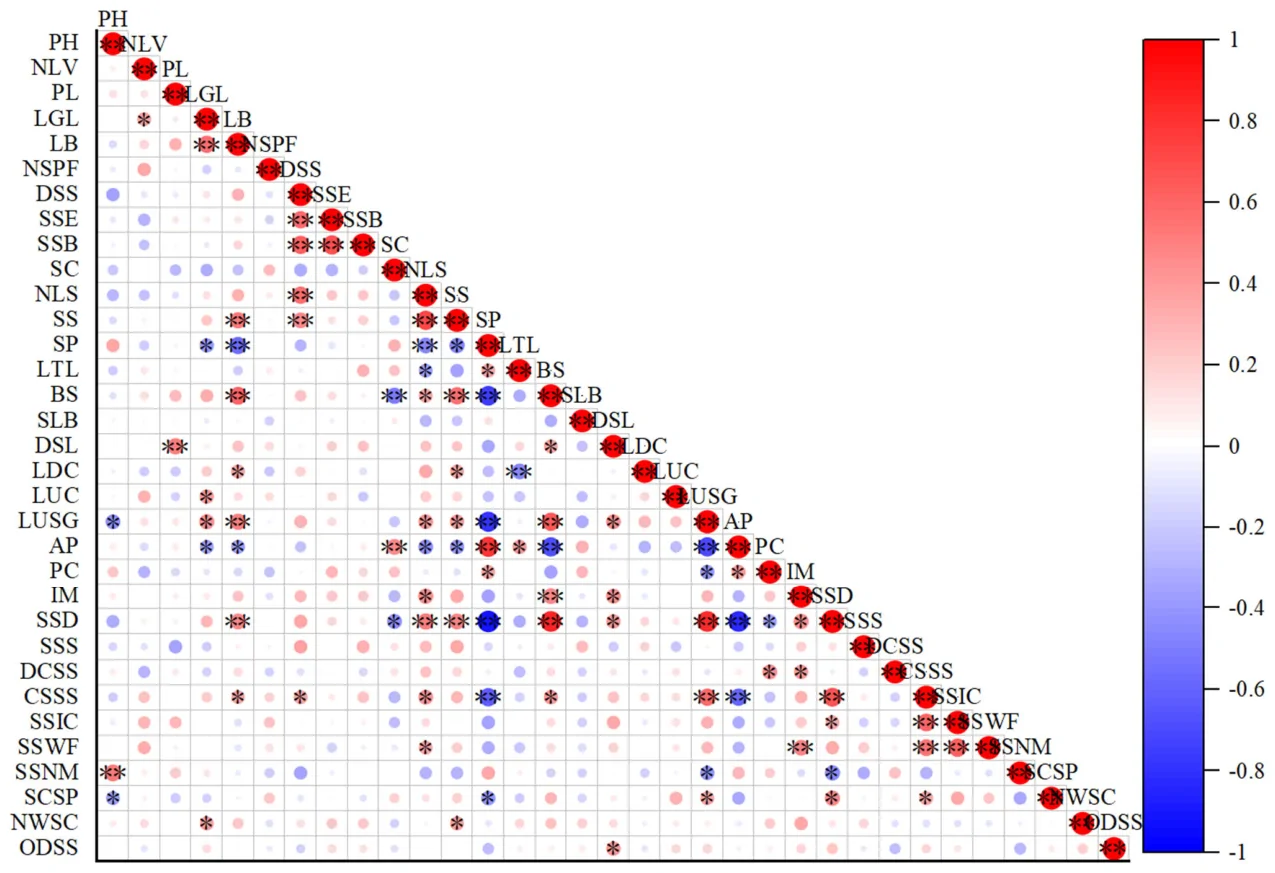
Correlation analysis of 33 morphological traits in *Hydrangea* germplasm. Note: PH, NLV, PL, LGL, LB, NSPF, DSS, SSE, SSB, SC, NLS, SS, SP, LTL, BS, SLB, DSL, LDC, LUC, LUSG, AP, PC, IM, SSD, SSS, DCSS, CSSS, SSIC, SSWF, SSNM, SCSP, NWSC, and ODSS represent plant height, logarithm of leaf lateral veins, petiole length, leaf length, leaf width, number of sepals per flower, sepal diameter per flower, length of sterile sepals, width of sterile sepals, stem color, number of stem lenticels, stem lenticel size, stem pubescence, leaf apex size, leaf shape, leaf base shape, leaf margin serration depth, leaf dominant color, leaf adaxial color, leaf adaxial gloss, leaf abaxial pubescence, petiole color, inflorescence shape, sterile sepal density, sterile sepal apex shape, degree of vesiculation of sterile sepals, cross-sectional shape of sterile sepals, inward curvature of sterile sepal margins, wrinkles on sterile sepals, dominant color of sterile sepals, number of whorls of sterile sepals, and degree of overlap of sterile sepals. * indicates a significant correlation at the 0.05 level; ** indicates a significant correlation at the 0.01 level. Red indicates a positive correlation; blue indicates a negative correlation. The darker the color, the stronger the correlation.

A correlation analysis was conducted on 33 morphological traits of *Hydrangea* germplasm from wild species and cultivated varieties (Figure 4). The results showed varying degrees of correlation among the different morphological traits. Among the 24 quality traits, 12 pairs exhibited highly significant correlations (*p* < 0.01). Specifically, the overlap of sterile sepals (SSWF) was highly significantly positively correlated with the notching of sterile sepals (SSIC) and inflorescence shape (IM); the shape of the leaf base (BS) was extremely significantly positively correlated with the glossiness of the upper leaf surface (LUSG) and extremely significantly negatively correlated with the pubescence on the lower leaf surface (AP); the dominant leaf color (LUC) was extremely significantly negatively correlated with the pubescence on the lower leaf surface (AP), while it was extremely significantly positively correlated with the logarithm of lateral veins (NLV). Conversely, the distinctiveness of fertile flowers in the inflorescence (SSNM) was significantly or extremely significantly negatively correlated with the density of sterile flowers (SSD) and leaf upper surface gloss (LUSG). This suggests that variation in leaf and floral organ traits may jointly influence the overall ornamental morphology of the inflorescence. Among the nine quantitative traits, plant height (PH) was extremely significantly negatively correlated with leaf upper surface gloss (LUSG) and extremely significantly positively correlated with the distinctiveness of fertile flowers in the inflorescence (SSNM); leaf length (LGL) was significantly or extremely significantly positively correlated with leaf width (LB), and petiole length (PL) was significantly or extremely significantly positively correlated with leaf width (LB). Furthermore, quantitative traits of floral organs—such as the diameter of a single sepal (DSS), the number of sepals per flower (NLS), sepal length (SSE), and sepal width (SSB)—were mostly highly significantly positively correlated, indicating a close synergistic developmental relationship among these traits. It is worth noting that sterile flower density (SSD) was extremely significantly positively correlated with leaf width (LB), but significantly or extremely significantly negatively correlated with plant height (PH) and the distinctiveness of fertile flowers in the inflorescence (SSNM), indicating a synergistic and constraining relationship between leaf growth and floral organ development. In future breeding programs, priority should be given to selecting germplasm with large leaves, good gloss, high sterile sepal overlap, and distinct marginal notches as parental lines for cultivation. Additionally, for parental lines intended for hybridization, germplasm exhibiting good coordination in plant height, the distinctiveness of fertile flowers in the inflorescence, and sterile flower density should be selected for propagation.

#### 2.2.3. Comprehensive Evaluation of *Hydrangea* Germplasm Resources

After coding and standardizing the 33 morphological traits of the test materials, the values were substituted into the factor score coefficients corresponding to the nine principal components. The functional expression for the principal component scores is defined as: F1 = −0.301 × 1 + 0.145 × 2 + 0.086 × 3 + … + 0.191 × 33; similar equations apply to F2 through F9. Subsequently, the principal component value (F) was derived using the formula: F = (0.2395F1 + 0.0989F2 + 0.0854F3 + 0.0763F4 + 0.0708F5 + 0.0554F6 + 0.0524F7 + 0.0466F8 + 0.0457F9)/0.770, where the coefficients represent the variance contribution rate of each principal component as a weighting factor. Finally, the germplasm was evaluated and ranked based on its F-value; a higher F-value indicates better overall morphological performance of the germplasm (Table 4). Six varieties achieved a composite score exceeding 5. Among them, *H. m.* ‘Bo da lan’ exhibited distinctive overall characteristics among the cultivated varieties: moderate plant vigor, broad leaves, sterile sepals arranged in a windmill pattern with wavy margins, high sepal overlap, and pure blue flower color. With excellent overall ornamental traits, it serves as an ideal parental material for breeding large-flowered, blue-colored *Hydrangea* varieties. At the same time, it stands out most prominently among *H. hypoglauca* germplasm, exhibiting typical characteristics such as papery leaves, a leaf underside densely covered with short soft hairs (giving the underside a grayish-white appearance), nearly conical inflorescences, and sterile sepals with finely serrated margins. Furthermore, its individual sepals are relatively large and possess high ornamental value, making it an important source material for genetic improvement and new variety breeding within the *Hydrangea* genus.

#### 2.2.4. Cluster Analysis of *Hydrangea* Germplasm Based on Morphological Traits

**Figure 5:**
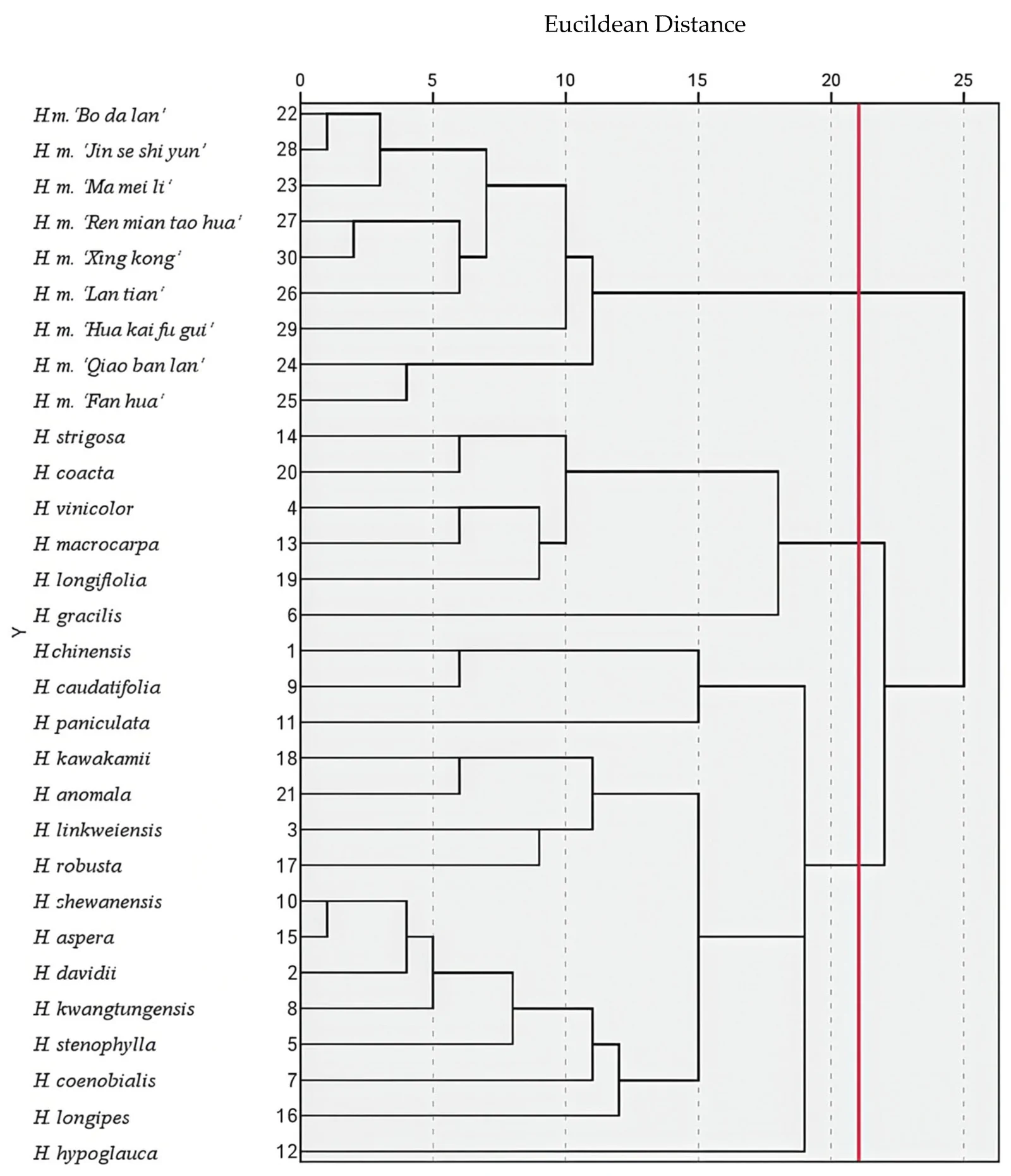
Cluster Analysis of 30 *Hydrangea* Germplasm Lines Based on Morphological Traits. Note: The solid red line indicates a threshold of 20.37 for the Euclidean distance, resulting in three clusters.

A cluster analysis was conducted by combining all morphological traits (Figure 5). The results indicate that, at an Euclidean distance of 20.37, the 30 *Hydrangea* accessions can be classified into three major clusters. Cluster I comprises nine cultivated varieties (22–30), namely *H. m.* ‘Bo da lan’, *H. m.* ‘Ma mei li’, *H. m.* ‘Qiao ban lan’, *H. m.* ‘Fan hua’, *H. m.* ‘Lan tian’, *H. m.* ‘Ren mian tao hua’, *H. m.* ‘Jin se shi yun’, *H. m.* ‘Hua kai fu gui’ and *H. m.* ‘Xing kong’. Their common characteristics include: a highly glossy upper leaf surface, glabrous stems, densely clustered sterile flowers, and sepals often exhibiting bubble-like protrusion or slight indentations; the flowers display a wide variety of colours (blue, pink and purple), reflecting the breeding trends in cultivated varieties towards large flowers, double blooms and diverse colours.

Cluster II comprises six wild species (4, 6, 13, 14, 19, and 20), namely *H. vinicolor*, *H. gracilis*, *H. macrocarpa*, *H. strigosa*, *H. longiflolia* and *H. coacta*. The common characteristics of this cluster are: the widespread presence of pubescence on the undersides of leaves or stems; a high frequency of secondary leaf colours (light green/medium green); stems that are predominantly brown or black (with black stems in the slender-stemmed hydrangea); and sterile sepals exhibiting a primary colour of pink or pale yellow, distinguishing it from Cluster III, where the sepals are pure white. Clade III comprises 15 wild species (1, 2, 3, 5, 7, 8, 9, 10, 11, 12, 15, 16, 17, 18, and 21), namely *H. chinensis*, *H. davidii*, *H. linkweiensis*, *H. stenophylla*, *H. coenobialis*, *H. kwangtungensis*, *H. caudatifolia*, *H. zhewanensis*, *H. paniculata*, *H. hypoglauca*, *H. aspera*, *H. longipes*, *H. robusta*, *Hydrangea kawakamii* and *H. anomala*. The key characteristics of this cluster are: leaf tip length is mostly ‘long’ or ‘medium’, the depth of serration along the leaf margin is generally ‘strong’ or ‘medium’, leaf shape is mostly ovate or narrowly elliptical, and all sterile sepals are white. The classification of the above three clades indicates that all cultivated varieties of *Hydrangea macrophylla* (Thunb.) Ser. (Clade I) are morphologically distinct from the wild species (Clades II and III), suggesting that artificial selection has resulted in the cultivated varieties forming an independent morphological unit; whereas the 21 wild accessions were further subdivided into two clusters, with Cluster III showing significant differentiation from Cluster II in terms of leaf shape, serration depth, and sepal colour esources.

### 2.3. Analysis of SSR Genetic Diversity in Hydrangea Germplasm

#### 2.3.1. Analysis of SSR Polymorphism

Using the 22 pairs of SSR markers selected (The first 16 primer pairs are newly designed; the latter 6 pairs are taken from reference [28]), a total of 30 allelic loci were amplified, with the number of allelic loci (Na) ranging from 8.0 to 33.0, averaging 20.59 (Table 5). Since the tested materials encompassed multiple wild species and cultivated varieties of the genus *Hydrangea*, representing a broad genetic background and significant interspecific genetic differentiation, the number of detected alleles (Na = 8–33) was higher than that reported in studies of closely related genera or the same species. This indirectly reflects the rich genetic diversity of the materials selected for this study. The number of effective alleles (Ne) ranged from 3.84 (STAB391) to 23.04 (P26), with an overall average of 13.49. The genetic diversity index (I) ranged from 1.65 to 3.30, with primer P26 showing the highest value and STAB391 the lowest, averaging 2.68. The observed heterozygosity (Ho) and expected heterozygosity (He) ranged from 0.167 (STAB321) to 0.933 (STAB391) and from 0.739 (STAB391) to 0.957 (P26), respectively, with mean values of 0.574 and 0.901. For most loci, the Ho value was lower than the corresponding He value, and the polymorphism information content (PIC) ranged from 0.710 to 0.979, with an average of 0.894. The PIC values for all primers exceeded the threshold of 0.50, indicating that these SSR markers exhibit high polymorphism in the *Hydrangea* materials tested and can be effectively used for subsequent genetic diversity analyses.

#### 2.3.2. Genetic Similarity Analysis

Using GenAIEX 6.4.1, we calculated genetic similarity coefficients to evaluate the *hydrangea* accessions. The genetic similarity coefficients of the tested accessions ranged from 0.015 to 0.730, with an average of 0.118. The lowest genetic similarity coefficient (0.015) was observed between accession 6 (*H. gracilis*) and accession 3 (*H. linkweiensis*), indicating the most significant genetic variation and the most distant phylogenetic relationship among these accessions. In contrast, the highest genetic similarity coefficient (0.730) was observed between accession 28 (*H. m.* ‘Jin se shi yun’) and accession 23 (*H. m.* ‘Ma mei li’), indicating that these two accessions are genetically the most closely related. The complete genetic similarity coefficient matrix is shown in Supplementary Materials.

#### 2.3.3. Cluster Analysis of *Hydrangea* Accessions Based on SSR Markers

The clustering results were converted into a cophenotypic matrix, and a Mantel test was performed to compare these matrices with the similarity matrix. The results of the Mantel test showed no significant correlation between the morphological similarity coefficient and the SSR genetic similarity coefficient (r = 0.003, *p* = 0.517), indicating that morphological traits and molecular markers reflect information from different dimensions when elucidating the phylogenetic relationships of Hydrangea germplasm. In the cluster analysis, the correlation between morphological traits and the clustering results (r = 0.8296) was higher than that for SSR markers (r = 0.532), suggesting that morphological traits and SSR markers are complementary rather than mutually exclusive in the evaluation of *Hydrangea* germplasm resources. This result may be related to the relatively stable genetic basis of morphological traits in *Hydrangea* species, or it may reflect that the patterns of variation in SSR markers at the interspecific level do not fully align with morphological differentiation. Furthermore, we analyzed the correlation between the similarity matrices derived from morphological and molecular data and found a low correlation (r = 0.003), indicating that morphological variation in *Hydrangea* is relatively independent of genetic differentiation at the DNA level.

**Figure 6:**
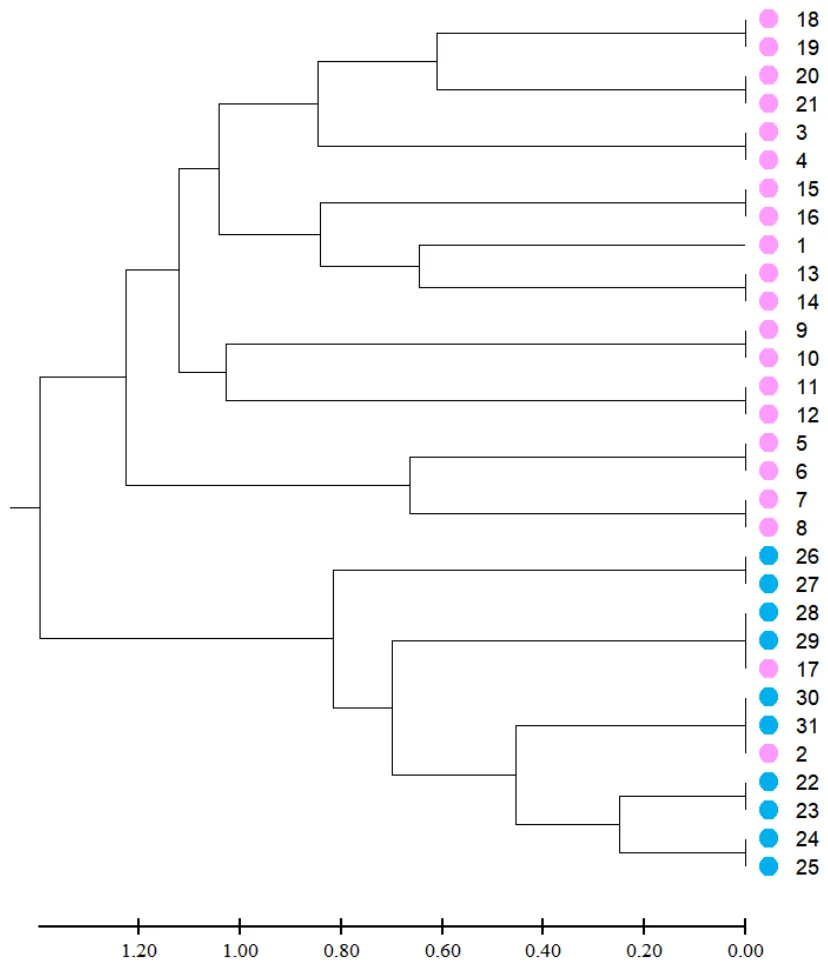
UPGMA clustering of Nei’s pairwise SSR genetic distances between 30 *Hydrangea* varieties. Note: The pink dots represent wild hydrangea species, whilst the blue dots represent cultivated varieties.

UPGMA cluster analysis showed (Figure 6) that the 22 pairs of SSR markers could effectively distinguish the tested materials. The 30 *Hydrangea* accessions were divided into two major clusters. Cluster I included 17 of the 21 wild *Hydrangea* species, with the exception of 2 (*H. davidii*) and 17 (*H. robusta*); Cluster II consisted primarily of cultivated varieties (22–31) and the two wild species 2 (*H. davidii*) and 17 (*H. robusta*). The results indicate that there is a relatively distinct genetic differentiation between wild species and cultivated varieties, and that SSR molecular markers can effectively distinguish between these two types of germplasm resources. The UPGMA dendrogram showed that the genetic material of wild *Hydrangeas* and cultivated varieties exhibited a relatively distinct separation on the clustering diagram, indicating that the clustering results are to some extent correlated with the origin of the genetic material (wild species/cultivated varieties). Among them, most wild *Hydrangeas* clustered into one branch, while all cultivated varieties clustered into another. However, two wild species (*H. davidii*) and one (*H. robusta*) were clustered with the cultivated varieties, suggesting that these wild species may be more closely related to the cultivated varieties. Overall, SSR molecular markers can effectively distinguish between wild and cultivated varieties of the genus *Hydrangea*, but the genetic relationships among some materials are relatively complex, which may be related to artificial selection and hybridization backgrounds.

#### 2.3.4. Construction of Fingerprint Profiles for 30 *Hydrangea* Samples

Based on the PIC (Polymorphism Information Content) of the primers, 8 pairs of core SSR markers were ultimately selected to construct fingerprint profiles for 30 Hydrangea samples. The fingerprint profiles have been deposited in the Science Data Bank and are available at https://www.scidb.cn/s/BbENBj (accessed on 8 September 2026). It is worth noting that when using the first two primer pairs, P4 and P5, 27 of the 30 *Hydrangea* samples could be identified, while 3 cultivated varieties could not be distinguished (*H. m.* ‘Bo da lan’, *H. m.* ‘Ma mei li’ and *H. m.* ‘Ren mian tao hua’). However, adding the P26 marker allowed for the identification of all 30 *Hydrangea* samples. This indicates that distinguishing among *Hydrangea* germplasm is relatively easy, whereas Cultivated varieties—all belonging to the large-leaved *Hydrangea* clusters—are more difficult to distinguish and require a greater number of markers.

## 3. Discussion

### 3.1. Morphological Diversity Characteristics of Hydrangea Germplasm

In this study, the 30 *Hydrangea* accessions exhibited rich morphological diversity in floral, leaf, and stem traits. Among these, the genetic diversity indices for floral traits (H′ = 0.124–1.559) were generally higher than those for vegetative organ traits, particularly for the dominant color of sterile sepals (SCSP, H′ = 1.559) and inflorescence shape (IM, H′ = 1.123). This is highly consistent with the breeding objectives for *Hydrangeas* as ornamental plants—artificial selection primarily targets ornamental traits such as flower form, flower color, and sterile calyx density, providing a rich genetic basis for directed improvement of flower color. Among vegetative organ traits, petiole length (CV = 70.76%), leaf width (CV = 46.31%), and plant height (CV = 40.76%) exhibited high coefficients of variation, and the H′ values for quantitative traits such as leaf length, leaf width, and plant height all exceeded 1.0, indicating strong potential for genetic improvement; The Pielou evenness index (E = 0.486–0.972) for quantitative traits was generally higher than that for qualitative traits (E = 0.179–0.948), indicating that quantitative traits exhibit a continuous and uniform gradient distribution among germplasm accessions, making them more suitable as quantitative indicators for directed selection. It is worth noting that the proportions of stem pubescence (93.3%) and leaf-underside pubescence (86.7%) were extremely high in the wild types, whereas cultivated varieties were all glabrous; this trait can serve as a reliable morphological marker for distinguishing wild types from cultivated varieties. Principal component analysis (PCA) further clearly separated the wild species from the cultivated varieties (with no overlap in the 95% confidence ellipses), indicating that long-term artificial selection has driven significant morphological differentiation in *Hydrangea* germplasm; Cluster II (e.g., *H. vinicolor*, *H. strigosa*, etc.) exhibits a transitional state from wild-type to cultivated-type in traits such as stem color and sepal color, possibly representing an intermediate evolutionary stage in the domestication process of *Hydrangeas*. This finding is consistent with the results of the morphological structural analysis of the *H. aspera* subspecies conducted by Morel et al. [12]. However, the survey of morphological traits was concentrated within a single growing season, failing to assess the impact of interannual environmental fluctuations on phenotypes. Furthermore, since only a single specimen was collected for some rare wild species, it is difficult to fully reflect the range of intraspecific variation. Future studies should be refined through repeated observations over multiple years and at multiple locations.

### 3.2. Genetic Diversity Levels of Hydrangea Germplasm

An analysis of the genetic diversity of 30 *Hydrangea* accessions using 22 pairs of SSR markers showed that both the average number of alleles (Na = 20.05) and the average expected heterozygosity (He = 0.901) were at relatively high levels, significantly higher than the results reported by Yamamoto et al. [16] for *H. robusta* (He = 0.48) and by Ito et al. [17] for *Hydrangea macrophylla* (He = 0.61). This indicates that the *Hydrangea* germplasm resources studied in this research possess high genetic diversity, which may be attributed to the fact that the tested materials encompassed multiple wild species and cultivated varieties with a broad genetic background. The polymorphism information content (PIC = 0.931) was extremely high, indicating that the selected SSR markers possessed high polymorphism and discriminatory power. Both UPGMA cluster analysis and principal component analysis (PCA) grouped the tested germplasm into two major clusters, corresponding to wild species and cultivated varieties, respectively, suggesting that the SSR markers can effectively distinguish *Hydrangea* wild species from cultivated varieties. Specimens 18–21 among the wild species clustered with the cultivated varieties, suggesting that these wild species may be closely related to the cultivated varieties or have undergone gene flow. The results of the Mantel test further validated the reliability of the cluster analysis (r = 0.532, *p* = 0.002), indicating that the clustering results were highly consistent with the original genetic data. The Mantel test comparing morphological and molecular data revealed no significant correlation between the two (r = 0.003, *p* > 0.05), suggesting that the morphological characteristics of *Hydrangea* species are relatively independent of genetic differentiation at the DNA level, and that morphological traits may be more susceptible to environmental influences. The cluster analysis and PCA results corroborated each other, indicating that SSR markers can effectively reveal the genetic relationships among *Hydrangea* germplasm, thereby providing an important molecular basis for the conservation, utilization, and breeding of *Hydrangea* germplasm resources. Chen et al. [29] have conducted karyotype analysis on the same germplasm used in this study, confirming the diploid nature of all materials (2n = 2x = 36), which provides a basis for the SSR diploidy scoring. Studies by Jones et al. [30] and Van Laere et al. [31] also indicate that ploidy variation in *Hydrangea* species is closely related to reproductive characteristics. It should be noted that, in our study, only a single sample was collected for some varieties, making it impossible to assess intraspecific genetic variation. This may have led to an underestimation of the level of diversity within the population. In future studies, the number of samples per variety should be increased (to at least 10–15 individuals) to more accurately reflect the true genetic structure.

### 3.3. Cluster Analysis Based on SSR Markers

Cluster analysis based on Nei’s genetic distance grouped the 30 *Hydrangea* accessions into two clusters (Figure 5). Since SSR markers exhibit high intraspecific polymorphism and may exhibit allelic heterogeneity, the UPGMA tree based on the SSR similarity matrix should be interpreted as a genetic clustering diagram rather than a strict phylogenetic tree. Therefore, this study does not use it to infer ancestral relationships among species but only to illustrate genetic similarity among accessions. The clustering results show that Cluster I consists mainly of wild species (such as *H. chinensis* and *H. linkweiensis*), while Cluster II consists mainly of cultivated varieties and includes some special wild species (such as *H. robusta* and *H. davidii*). Some wild species (such as *H. robusta* and *H. davidii*) are clustered together with cultivated varieties, suggesting that these wild species may have been involved in the domestication process of modern *Hydrangea* varieties; however, this requires further validation through sequence-based molecular phylogenetics (such as chloroplast DNA or nuclear gene sequences) [32]. The co-phenotypic correlation coefficient was used to assess the fit of the clustering trees to the original distance matrix [33]; the co-phenotypic correlation coefficient for the morphological clustering tree was 0.8296, while that for the SSR clustering tree was 0.532, indicating that both clustering trees could reasonably well reflect the original distance information, with the morphological clustering tree exhibiting a higher degree of fit. Yang et al. [7] updated the subgenus classification of the Hydrangeaceae, providing the taxonomic background for the taxonomic grouping in this study.

### 3.4. Analysis of Consistency Between Morphological and Molecular Data

To assess the consistency between morphological traits and SSR molecular markers in revealing genetic relationships among germplasm, Mantel tests were conducted on the morphological similarity coefficient matrix and the SSR genetic similarity coefficient matrix. The results showed an extremely low and nonsignificant correlation between the two (r = 0.003, *p* = 0.517), indicating a lack of consistency between patterns of morphological diversity and molecular genetic diversity. This reflects that morphological traits and molecular markers convey different biological information regarding the phylogenetic relationships of *Hydrangea* germplasm. This result is consistent with the trend reported in previous studies [34] (r ≈ 0.20), both indicating a weak correlation; however, the correlation coefficient in this study was lower. This extremely low correlation may stem from the fact that morphological traits are easily influenced by environmental conditions (light, moisture, soil pH, etc.) and developmental stages, exhibiting strong phenotypic plasticity, whereas SSR markers directly reflect variation at the genomic DNA level and are unaffected by the environment. The morphological traits selected in this study were primarily floral and foliar traits related to ornamental value, which may have been subject to strong artificial selection pressure, whereas SSR markers are neutral markers; thus, the two are subject to different selection pressures. Frequent interspecific hybridization and gene flow may occur in *Hydrangea* species, leading to an incomplete coupling between morphological characteristics and genetic background. Furthermore, since only a single sample was collected for some accessions in this study, it is difficult to assess intraspecific variation, which may also affect the accuracy of the morphological–molecular correlations. Future studies should combine morphological traits with genomic data (such as SNPs) to conduct genome-wide association studies in order to identify candidate genes controlling key ornamental traits. In summary, although no significant correlation was observed between morphological and molecular data in this study, this does not negate the value of using the two in combination. Morphological traits and molecular markers provide information at the phenotypic and genotypic levels respectively; the former reflect the ornamental characteristics and environmental adaptability of germplasm, whilst the latter reveal genetic background and phylogenetic relationships. The combination of the two enables a comprehensive evaluation of *Hydrangea* germplasm resources from different dimensions, providing a more complete basis for germplasm identification, conservation and breeding.

### 3.5. Limitations and Future Directions

In this study, only a single sample was collected for some varieties, making it difficult to assess the level of intraspecific genetic variation. Furthermore, the survey of morphological traits was confined to a single growing season and did not account for interannual variation. Although SSR markers exhibit high polymorphism, they are not suitable for inferring deep-level phylogeny. Future research should increase the number of samples, conduct repeated observations over multiple years, include more international varieties, and perform true molecular phylogenetic analyses based on sequence markers to further refine the evaluation system for *Hydrangea* germplasm resources.

## 4. Materials and Methods

### 4.1. Experimental Materials

This study utilised 30 accessions of *Hydrangea* species from China (Table 6), of which 21 wild-collected accessions were sourced from wild distribution areas in China (including Yunnan, Guizhou, Sichuan, Hubei, Jiangxi, Shaanxi, Taiwan, Zhejiang, Anhui, Hunan, Guangxi, Guangdong and Gansu); all wild accessions were identified and confirmed in accordance with the *Flora of China*. The remaining nine accessions were cultivated varieties, used as controls. All germplasm materials were uniformly transplanted to the Nanguo Mountain Flowers Base in Kunming, Yunnan Province (24.86° N, 102.98° E) for cultivation within the same garden, and samples were collected uniformly in August 2024.

### 4.2. Experimental Methods

#### 4.2.1. Study of Morphological Traits

Under identical habitat conditions and based on the plant growth status, 10 vigorous plants with consistent growth patterns were selected from each germplasm accession at the Nanguo Mountain Flowers Base in Yuxi City, Yunnan Province, in August 2024 for the assessment of morphological traits. Each plant was measured three times to ensure data accuracy. This period was conducive to comprehensive observation and statistical analysis of various morphological characteristics. A total of 33 morphological traits were recorded in situ from these 30 germplasm accessions, including 24 qualitative traits (Nos. 1–25) and 9 quantitative traits (Nos. 26–34) (Table 7), with repeated measurements taken of the inflorescences and mature leaves for each trait. This method ensured a comprehensive assessment of morphology. Among these, 15 traits were related to floral characteristics, including 11 qualitative traits (inflorescence shape, sterile flower density, apical shape of sterile sepals, degree of vesiculation of sterile sepals, cross-sectional shape of sterile sepals, inward curvature of sterile sepal margins, wrinkling of sterile sepals, notches on the margins of sterile sepals, dominant color of sterile sepals, number of whorls of sterile sepals, and degree of overlap of sterile sepals), as well as 4 quantitative traits (number of sepals per flower, diameter of a single sepal, length of sterile sepals, and width of sterile sepals). In addition, there are 13 leaf-related traits: logarithm of lateral veins, petiole length, leaf length, leaf width, leaf apex length, leaf shape, leaf base shape, serration depth, dominant leaf color, leaf adaxial color, leaf adaxial gloss, leaf abaxial pubescence, and petiole color; as well as 4 stem-related traits: stem color, number of stem lenticels, stem lenticel size, stem pubescence, and plant height.

#### 4.2.2. DNA Extraction and SSR Analysis

Genomic DNA was extracted from *Hydrangea* samples using a commercial kit. The kit used was the Ezup Column-Based Plant Genomic DNA Extraction Kit (Shanghai Geneworks Biotechnology Co., Ltd., Shanghai, China). DNA purity was assessed by 1.5% agarose gel electrophoresis, and DNA concentration was measured using a NanoDrop 2000 spectrophotometer (Thermo Fisher Scientific, Wilmington, DE, USA). All samples were diluted to 100 ng/μL and stored for later use.

The PCR amplification system consisted of: 0.5 µL dNTP (mix), 0.2 µL Taq enzyme (Shanghai Genework Bioengineering Co., Ltd.), 2.5 µL Taq Buffer (with MgCl_2_), 0.5 µL forward fluorescent primer (10 µM), 0.5 µL reverse primer (10 µM), 1 µL genomic DNA (20 ng/µL), and ddH_2_O added to a total volume of 25 µL. The PCR reaction program is as follows: 5 min pre-denaturation at 95 °C; 30 s denaturation at 94 °C; 30 s annealing at 58 °C; 30 s extension at 72 °C; 10 cycles of steps 2–4; 30 s denaturation at 94 °C; 30 s annealing at 58 °C; 30 s extension at 72 °C; 30 cycles of steps 6–8; and 10 min repair extension at 72 °C.

PCR products were analyzed via 2% agarose gel electrophoresis and 8% PAGE gel electrophoresis to detect polymorphisms and determine the annealing temperature, followed by fluorescence capillary electrophoresis.

### 4.3. Data Processing and Statistical Analysis

#### 4.3.1. Morphological Analysis

Microsoft Excel 2019 was used to collect, organize, and classify morphological data. Qualitative traits were quantified and coded, and the range and frequency distribution of each trait were calculated. For quantitative traits, a four-level classification was applied. The criteria are as follows: Class 1: X < S − SD; Class 2: S − SD ≤ X < S; Class 3: S ≤ X < S + SD; Class 4: X ≥ S + SD (where X is the measured value, S is the mean, and SD is the standard deviation). This classification method converts continuous quantitative traits into ordinal data, facilitating the calculation of the Shannon–Wiener diversity index (H′) and the Pielou evenness index (E). Subsequently, statistical parameters were calculated, including the mean (S), standard deviation (SD), maximum (Max), minimum (Min), coefficient of variation (CV), Simpson’s index (D), Pielou’s evenness index (E) and the Shannon–Wiener diversity index (H′). The formula for the Simpson index D is D = 1 − ∑ (Pi^2^) [35], the Pielou evenness index E is calculated as E = H′/ln k [36], and the genetic diversity index H′ is calculated as H′ = −∑ Pi × ln(Pi), where Pi represents the percentage of the sample relative to the total population, and ln denotes the natural logarithm [37]. The Shannon–Wiener index (H′), Simpson’s index (D) and Pielou’s evenness index (E) used in this study are all employed to quantify the level of phenotypic diversity in morphological traits, as distinct from the genetic diversity revealed by the subsequent SSR molecular markers; the two are clearly distinguished in terms of both concept and level. Following this standardization process, correlation analysis was conducted using Origin 2021 software [38]. Heatmaps were generated at significance levels of 0.01 and 0.05. Principal component analysis (PCA) of morphological traits was performed using SPSS 27.0 software [39]. Principal components were identified based on eigenvalues and cumulative contribution rates; eigenvector coefficients were calculated, and scoring function equations were established. In the comprehensive evaluation of principal components, the variance contribution ratio (λ_i_) of each principal component is used as a weight. The formula for calculating the composite score F is F = (λ_1_F_1_ + λ_2_F_2_ + … + λ_n_F_n_)/Σλ_i_, where F_i_ represents the score for each principal component, and the denominator Σλ_i_ represents the cumulative contribution ratio of the selected principal components (expressed as a decimal). This standardization process ensures that the composite score has the same units as the original principal component scores, thereby facilitating comparisons among different germplasm resources. A model for evaluating the morphological traits of Hydrangeas was constructed by calculating the f-value [40], which represents the weighted contribution of each principal component to the variance. Cluster analysis was performed using Origin 2021 software, the UPGMA method, and Euclidean distance to construct a clustering tree.

#### 4.3.2. SSR Analysis

GenAIEx 6.5 software was used to analyze genetic diversity, calculating indicators such as the number of alleles (Na), effective number of alleles (Ne), observed heterozygosity (Ho), and expected heterozygosity (He) for each population. MEGA 11.0.13 (Molecular Evolutionary Genetics Analysis, version 11) was then used to perform UPGMA cluster analysis based on Nei’s genetic distance. Ceruvs 3.0.7 software was used to analyze the Polymorphism Information Content (PIC) of the primers in *Hydrangea*. The Mantel test was employed to assess the correlation between the morphological distance matrix and the SSR genetic distance matrix. Using GenAIEx 6.5 software, the Gower distance was set as the morphological dissimilarity matrix, and Nei’s genetic distance was set as the molecular dissimilarity matrix, with a significance level of 999 permutations. Based on a comprehensive analysis combining genetic diversity data such as Na and Ne, the optimal primer pair was selected to construct molecular identification codes for *Hydrangeas* [41]. A QR code generator (https://www.hlcode.cn/) was used to generate scannable QR codes for *Hydrangea* DNA identification codes by incorporating relevant information about the *Hydrangeas*.

## 5. Conclusions

This study comprehensively employed morphological trait surveys and SSR molecular marker technology to conduct a systematic analysis of the genetic diversity, phylogenetic relationships, and consistency between morphological and molecular data of 30 *Hydrangea* accessions (21 wild species and 9 cultivated varieties). The results indicate that *Hydrangea* germplasm exhibits rich diversity, with diversity in floral traits (e.g., the dominant color of sterile sepals, H′ = 1.536) being higher than that in vegetative organ traits. Petiole length showed the greatest variation (CV = 72.65%). Principal component analysis clearly separated wild species from cultivated varieties, revealing phenotypic differentiation driven by artificial selection; Sixteen pairs of highly polymorphic SSR markers (average PIC = 0.874) were successfully developed. Combined with six previously published primer pairs, a total of 22 markers were used for genetic evaluation, revealing a moderate level of genetic diversity (average Na = 20.59, He = 0.901, PIC = 0.874). Cluster analysis grouped the germplasm into two clusters, with cultivated varieties forming a distinct branch. The SSR fingerprinting profile constructed using 8 pairs of core primers enables the accurate identification of 30 accessions. The Mantel test revealed no significant correlation between the morphological similarity coefficient and the SSR genetic similarity coefficient (r = 0.003, *p* = 0.517), indicating that morphological traits and molecular markers reflect different dimensions of information in elucidating the phylogenetic relationships of *Hydrangea* germplasm; the two types of data are complementary rather than interchangeable. Therefore, the combined use of morphological traits and molecular markers in the evaluation of *Hydrangea* germplasm resources can provide more comprehensive information. This study provides a theoretical basis and technical support for the identification, conservation, and molecular marker-assisted breeding of *Hydrangea* germplasm.

## Figures and Tables

**Table 1:** Phenotypic Diversity of 24 Quality Traits in *Hydrangea* Germplasm.

Morphological Characteristics	Frequency (%)						H′	D	E
	1	2	3	4	5	6			
SC	70.00	26.67	3.33				0.716	0.562	0.651
NLS	63.33	20.00	6.67	10.00			0.995	0.456	0.718
SS	76.67	20.00	3.33				0.639	0.629	0.582
SP	33.33	66.67					0.637	0.556	0.918
LTL	66.67	16.67	16.67				0.868	0.500	0.790
BS	13.33	50.00	3.33	23.33	3.33	6.67	1.362	0.329	0.760
SLB	83.33	3.33	6.67	6.67			0.626	0.704	0.452
DSL	13.33	26.67	53.33	6.67			1.137	0.378	0.820
LDC	3.33	33.33	63.33				0.769	0.513	0.700
LUC	10.00	90.00					0.325	0.820	0.469
LUSG	63.33	36.67					0.657	0.536	0.948
AP	36.67	63.33					0.657	0.536	0.948
PC	76.67	6.67	16.67				0.683	0.620	0.622
IM	56.67	20.00	16.67	6.67			1.123	0.393	0.810
SSD	66.67	3.33	30.00				0.745	0.536	0.678
SSS	30.00	70.00					0.611	0.580	0.881
DCSS	86.67	13.33					0.393	0.769	0.567
CSSS	83.33	16.67					0.451	0.722	0.650
SSIC	86.67	13.33					0.124	0.769	0.179
SSWF	90.00	3.33	6.67				0.389	0.816	0.354
SSNM	46.67	46.67	6.67				0.892	0.440	0.812
SCSP	43.33	6.67	16.67	6.67	13.33	13.33	1.559	0.260	0.870
NWSC	93.33	6.67					0.245	0.876	0.353
ODSS	3.33	33.33	63.33				0.769	0.513	0.700

Note: For the full names and coding standards corresponding to the trait abbreviations, see Section 4.2.1. The frequencies (%) 1–6 indicate the distribution proportions of the different state levels for each trait; H′ is the Shannon–Wever diversity index, D is the Simpson index, and E is the Pielou evenness index.

**Table 2:** Phenotypic Diversity of 9 Quantitative Traits in *Hydrangea* Germplasm.

Morphological Characteristics	Maximum Value	Minimum Value	Average	Standard Deviation	Range	CV%	H′	E
PH/m	2.60	0.30	1.53	0.62	2.30	40.76	1.267	0.914
NLV/pair	10.00	5.00	7.23	1.36	5.00	18.75	1.068	0.972
PL/cm	6.00	0.30	1.98	1.40	5.70	70.76	0.840	0.606
LGL/cm	21.20	3.00	12.03	4.12	18.20	34.22	1.235	0.891
LB/cm	10.20	1.70	4.97	2.30	8.50	46.31	1.263	0.911
NSPF/pieces	6.00	4.00	4.20	0.48	2.00	11.53	0.534	0.486
DSS/cm	4.00	0.66	2.28	0.72	3.34	31.61	1.263	0.911
SSE/cm	2.50	0.28	1.30	0.48	2.23	37.20	1.137	0.820
SSB/cm	2.10	0.33	1.23	0.44	1.77	35.81	1.223	0.883

Note: For the full names corresponding to the trait abbreviations, see Section 4.2.1. CV denotes the coefficient of variation, H′ denotes the Shannon–Weaver diversity index, and E denotes the Pielou evenness index.

**Table 3:** Principal Component Analysis of 33 Morphological Traits in *Hydrangea* Germplasm.

Morphological Characteristics	PC1	PC2	PC3	PC4	PC5	PC6	PC7	PC8	PC9
SC	−0.301	0.044	0.469	−0.307	0.194	−0.221	0.408	−0.078	−0.153
NLS	0.145	−0.543	0.275	0.051	−0.291	0.11	0.504	0.068	0.334
SS	0.086	−0.039	0.685	0.092	−0.015	−0.257	−0.248	0.242	0.068
SP	0.402	0.006	0.373	−0.345	−0.492	0.293	0.184	−0.178	0.145
LTL	0.594	0.225	0.220	−0.34	−0.283	0.062	−0.082	0.233	0.278
BS	0.042	−0.449	−0.077	0.338	0.102	0.074	0.012	0.091	0.469
SLB	0.491	0.573	−0.229	0.236	−0.116	−0.217	0.085	−0.21	0.292
DSL	0.235	0.646	0.160	0.251	−0.005	0.098	−0.175	−0.476	0.024
LDC	0.177	0.657	0.096	0.55	−0.041	−0.129	0.024	−0.194	0.173
LUC	−0.437	−0.18	−0.379	0.127	0.164	0.441	−0.129	0.256	0.289
LUSG	0.668	0.275	−0.268	−0.071	0.344	−0.057	−0.029	−0.09	0.211
AP	0.663	0.312	−0.16	−0.212	0.097	0.085	0.076	0.21	0.263
PC	−0.893	0.146	0.032	0.060	0.106	−0.059	0.046	−0.07	0.151
IM	−0.328	0.037	0.236	0.600	−0.309	0.185	−0.096	0.100	0.244
SSD	0.800	0.026	0.244	−0.171	−0.024	−0.107	−0.13	0.301	−0.053
SSS	−0.288	0.239	−0.15	0.12	−0.283	0.088	0.364	0.278	−0.305
DCSS	0.404	0.038	0.520	0.338	0.234	0.127	−0.284	0.217	0.01
CSSS	0.299	0.141	−0.372	−0.469	−0.134	−0.051	−0.01	−0.163	−0.011
SSIC	0.262	−0.23	0.021	−0.284	0.048	0.571	0.11	−0.503	0.193
SSWF	0.845	−0.15	0.009	0.037	−0.163	0.12	−0.244	0.006	0.002
SSNM	−0.812	0.191	0.083	0.226	0.086	0.13	−0.059	0.084	0.182
SCSP	−0.339	0.47	0.102	−0.029	0.446	0.43	0.199	−0.111	−0.102
NWSC	0.503	0.325	0.254	0.098	0.46	0.093	0.294	0.232	−0.096
ODSS	0.939	−0.09	−0.007	0.048	−0.004	−0.038	−0.13	0.077	−0.198
PH	0.191	0.314	−0.572	0.16	−0.086	−0.148	0.377	0.353	0.021
NLV	−0.02	0.203	0.129	−0.41	0.712	0.153	−0.188	0.205	0.141
PL	0.733	−0.217	−0.037	0.268	0.079	−0.135	0.168	−0.219	0.022
LGL	0.401	−0.462	0.221	0.427	0.257	−0.168	0.179	−0.235	−0.063
LB	0.416	−0.298	−0.04	0.203	0.486	−0.123	0.524	−0.010	0.009
NSPF	−0.411	0.016	0.553	−0.294	0.164	−0.176	0.081	−0.134	0.216
DSS	0.379	−0.376	−0.298	0.17	0.272	0.396	−0.140	0.013	−0.103
SSE	0.182	0.385	0.272	0.028	−0.264	0.448	0.378	0.257	−0.019
SSB	0.191	0.038	0.197	0.29	−0.096	0.415	−0.056	−0.074	−0.591
Eigenvalue	7.904	3.262	2.820	2.518	2.335	1.795	1.729	1.538	1.508
Variance Contribution Rate%	23.951	9.886	8.544	7.632	7.076	5.441	5.238	4.660	4.570
Cumulative Contribution Rate%	23.951	33.837	42.381	50.013	57.088	62.529	67.767	72.427	76.998

Table note: For the full names and coding standards corresponding to the trait abbreviations, see Section 4.2.1. PC1–PC9 denote the first to ninth principal components, respectively. Loadings indicate the degree of contribution of each trait to the corresponding principal component; positive values indicate a positive correlation, whilst negative values indicate a negative correlation; values in bold indicate that the trait has a high loading on the corresponding principal component (absolute value > 0.6). Eigenvalues reflect the weight of each principal component in explaining variation; the variance contribution and cumulative contribution are expressed as percentages (%).

**Table 4:** Ranking of 30 *Hydrangea* accessions based on calculated correlations for relevant traits.

Variety	F1	F2	F3	F4	F5	F6	F7	F8	F9	F	Ranking
1	2.98	4.07	−1.88	4.30	2.32	4.81	−0.49	0.04	1.12	2.26	17
2	1.87	−0.36	2.34	2.73	3.08	4.35	1.44	1.33	0.80	1.89	22
3	2.18	0.71	5.12	4.49	4.49	3.65	−0.27	1.05	0.20	2.51	15
4	1.72	4.47	0.98	2.06	4.88	5.52	0.49	−1.64	−1.37	2.12	19
5	0.47	−1.32	−0.07	−2.17	3.33	3.80	2.19	1.46	−2.32	0.43	30
6	0.06	−4.73	−3.29	4.35	4.88	7.35	0.40	2.91	1.62	0.75	28
7	3.78	−1.08	4.72	0.75	1.24	5.82	0.28	2.51	0.84	2.39	16
8	3.46	2.11	0.15	−0.20	2.38	2.49	3.11	−0.22	1.03	2.00	21
9	7.28	6.35	−1.45	1.94	3.61	2.91	0.27	−0.53	1.82	3.75	12
10	2.13	−0.13	1.17	−1.72	2.03	3.89	2.12	0.89	0.16	1.28	26
11	4.55	6.11	−1.39	1.93	8.75	5.21	1.80	1.19	−0.54	3.58	13
12	4.24	7.27	4.29	4.08	2.89	6.20	3.80	1.11	−0.07	4.17	9
13	−0.30	3.10	−2.09	2.90	4.10	6.46	2.18	1.04	−1.88	1.30	25
14	0.50	−0.35	−0.62	0.54	6.48	7.19	−0.36	1.57	−0.76	1.23	27
15	3.66	0.93	0.77	0.16	1.05	5.01	2.42	2.32	−0.10	2.12	20
16	1.03	2.51	−0.63	1.67	2.56	2.40	1.05	3.32	−1.57	1.32	24
17	5.52	3.30	7.02	−0.09	5.38	3.16	0.87	2.59	0.54	3.88	10
18	0.88	3.99	2.11	3.68	3.89	3.29	0.48	1.80	0.73	2.17	18
19	0.64	0.87	−0.68	4.62	2.58	5.54	1.19	1.34	−0.60	1.46	23
20	−0.72	−0.46	−1.79	2.69	6.43	5.80	0.08	1.03	−2.08	0.74	29
21	5.22	4.42	1.75	4.05	4.49	2.77	0.32	2.50	−1.45	3.49	14
22	15.28	2.41	1.64	0.49	2.92	6.02	0.19	3.42	−0.28	6.20	2
23	14.79	2.82	0.36	−0.12	5.83	5.05	−0.90	3.09	−0.36	5.99	4
24	10.74	−0.65	1.63	3.86	6.00	4.85	2.26	1.36	−1.20	4.88	7
25	13.10	−2.44	0.17	2.18	5.81	4.70	2.83	3.17	1.06	5.32	5
26	10.23	0.30	−0.47	4.13	4.20	6.27	−1.20	0.73	−1.09	4.31	8
27	11.99	2.95	0.68	1.64	3.15	4.86	−1.33	1.93	−1.08	4.95	6
28	14.97	3.71	1.23	1.41	2.36	4.47	0.29	2.03	0.42	6.12	3
29	15.35	5.46	0.51	2.70	3.80	4.33	−0.26	0.78	1.21	6.56	1
30	9.50	−0.18	1.18	1.03	4.00	5.09	−0.49	1.84	−1.97	3.86	11

**Table 5:** 22 Analysis of Genetic Diversity of SSR Markers.

Tag Name	Primer Sequences (5′-3′)	Na	Ne	I	Ho	He	PIC
P1	F:TTTAGGAGTCCTTGGGTTTGGT R:TGGGTGCTTTTGTTTTCTACG	23	13.675	2.900	0.483	0.927	0.971
P4	F:ATCGACTCATCCAAGTATGCAGA R:CCACGTTTTCTACGGTAGCAGTA	22	15.574	2.935	0.552	0.936	0.974
P5	F:CCGATGTAGTGTTGGACCAATC R:TATGCGAAGAACGATGGTGTG	26	12.162	2.898	0.533	0.918	0.974
P7	F:AGCTCGAAGAAGATGGAGATTG R:CGCGCAATGTCATACTTGGA	27	20.024	3.154	0.690	0.950	0.976
P8	F:GGCTAATGCTCACTCTCTTTTCA R:GGGTAGAAAGGTAACAGGGAGGT	17	9.045	2.505	0.433	0.889	0.972
P11	F:CCCAAAAGAAACCTTACCCAAT R:AGGATTTAACCGCTAGATCTTCTC	15	7.407	2.344	0.800	0.865	0.971
P15	F:GTCGAAGGATCGGACGATTAC R:CGGTCCAGGTCCACTAATAAAC	23	18.225	3.032	0.593	0.945	0.970
P16	F:TCGAGTTCCATTGATTGTTCAAG R:ACCATGCAGTACTCTGTGCTAGTT	25	17.521	3.054	0.552	0.943	0.974
P17	F:GTTGTAAAGCATATAAAGGACCGA R:CTATTACCCCACCCAATGATAAA	20	13.235	2.802	0.633	0.924	0.974
P18	F:CCATGCTTCTCATCCTTTACAGT R:ACTTCAGCACGAGCATGAAATC	21	12.162	2.762	0.500	0.918	0.972
P19	F:GCAAAAAGAGTGGTGCATTTAGT R:TCGAAAGTCTTGTTGATTCCTCA	26	19.558	3.125	0.724	0.949	0.975
P24	F:CGAGTTCGGATTTCCTCTGGT R:AGTCCAATCTCAACACATTTTGC	15	7.792	2.350	0.367	0.872	0.966
P25	F:TGACCCTCCTGGGAAATATAAGT R:CCCATTTAAACCCAAAACTGTC	33	20.690	3.290	0.800	0.952	0.979
P26	F:AGGATGGTGAAGTTCTCTGACAA R:ACAATGCAACACTTGCAGGATT	31	23.041	3.297	0.655	0.957	0.975
P28	F:TCTACAATTCTGCTTTCACCTCAT R:CAGCCTATACAATCACATTCCACT	27	15.517	3.051	0.733	0.936	0.976
P29	F:ATTTCCTACGGTTGCATTGCTAT R:GACACAAGAAATCTTCAGGAGAGAG	24	15.517	2.959	0.633	0.936	0.973
STAB391-392	F:CCAACCCTTTCCTAAACTGCTCTT R:AAGGGTGTGTTTGAGGATGTTGAT	8	3.838	1.647	0.933	0.739	0.710
STAB321-322a	F:CAATTTCACCCATTTGAGGCC R:GGACTTACAGTCGCCGAGC	9	4.306	1.825	0.167	0.768	0.747
A029-A030b	F:CCTAATCTCCTCACTCTCTTTC R:GCATTGGACGGACTCAATTGG	19	9.677	2.590	0.433	0.897	0.890
STAB167-168	F:AATTAAAGCAAGGGAACAACAGCA R:GCTGCTGAGCGTGTCTTTGTAATA	10	4.675	1.859	0.200	0.786	0.759
STAB413-414	F:TCCCTCATTGATAAAGATGATGTACG R:GTGTTGCTGATGTTGCTGGTG	12	9.278	2.348	0.500	0.892	0.882
STAB421-422	F:GTCGAGGATTTCTTCTGCAAAACT R:ACATGTTGTTTCCGGTGTAATTGA	23	14.173	2.867	0.767	0.929	0.925
Mean		20.59	13.49	2.68	0.574	0.901	0.894

Note: Na (number of alleles); Ne (effective number of alleles); I (genetic diversity index); Ho (observed heterozygosity); He (expected heterozygosity); PIC (polymorphism information content); P1–P29 (the first 16 pairs) are primers newly developed for this study; the remaining six pairs are taken from reference [28].

**Table 6:** Information on the 30 *Hydrangea* Accessions Tested.

Number	Variety	Categories
1	*H. chinensis.* Maxim	wild accession
2	*H. davidii* Franch	wild accession
3	*H. linkweiensis* Chun	wild accession
4	*H. vinicolor* Chun	wild accession
5	*H. stenophylla* Merr. & Chun	wild accession
6	*H. gracilis* W. T. Wang & M. X. Nie	wild accession
7	*H. coenobialis* Chun	wild accession
8	*H. kwangtungensis* Merr.	wild accession
9	*H. caudatifolia* W. T. Wang & M. X. Nie	wild accession
10	*H. zhewanensis* P. S. Hsu & X. P. Zhang	wild accession
11	*H. paniculata* Siebold	wild accession
12	*H. hypoglauca* Rehder	wild accession
13	*H. macrocarpa* Hand.-Mazz	wild accession
14	*H. strigosa* Rehder	wild accession
15	*H. aspera*	wild accession
16	*H. longipes* Franch	wild accession
17	*H. robusta* Hook. f. & Thomson	wild accession
18	*Hydrangea kawakamii* Hayata	wild accession
19	*H. longiflolia* Hayata	wild accession
20	*H. coacta* C. F. Wei	wild accession
21	*H. anomala* D. Don	wild accession
22	*H. m.* ‘Bo da lan’	Cultivated Variety
23	*H. m.* ‘Ma mei li’	Cultivated Variety
24	*H. m.* ‘Qiao ban lan’	Cultivated Variety
25	*H. m.* ‘Fan hua’	Cultivated Variety
26	*H. m.* ‘Lan tian’	Cultivated Variety
27	*H. m.* ‘Ren mian tao hua’	Cultivated Variety
28	*H. m.* ‘Jin se shi yun’	Cultivated Variety
29	*H. m.* ‘Hua kai fu gui’	Cultivated Variety
30	*H. m.* ‘Xing kong’	Cultivated Variety

**Table 7:** Morphological Trait Indices and Standards for *Hydrangea* Germplasm.

Morphological Characteristics	Code	Documentation Standards
Stem Color	SC	Green = 1, Brown = 2, Black = 3
Number of lenticels on the stem	NLS	None or very few = 1, few = 2, moderate = 3, many = 4
Stem Lenticel Size	SS	Small = 1, Medium = 2, Large = 3
Stem is hairy	SP	None = 1, Yes = 2
Leaf Tip Length	LTL	None or short = 1, medium = 2, long = 3
Blade Shape	BS	Oval = 1, narrow oval = 2, elliptical = 3, broad oval = 4, round = 5, obovate = 6
Shape of the Leaf Base	SLB	Wedge-shaped = 1, Blunt-shaped = 2, Round = 3, Heart-shaped = 4
Depth of leaf margin serrations	DSL	None or very weak = 1, moderate = 2, strong = 3, very strong = 4
Leaf Color	LDC	Light green = 1, medium green = 2, dark green = 3
Color of the underside of the leaf	LUC	White = 1, Green = 2
Glossiness of the upper leaf surface	LUSG	None or weak = 1, strong = 2
The underside of the leaf is hairy	AP	None = 1, Yes = 2
Leaf stalk color	PC	Green = 1, Red = 2, Greenish-brown = 3
Inflorescence Shape	IM	Flat = 1, flattened spherical = 2, spherical = 3, nearly conical = 3
Density of Sterile Sepals	SSD	Sparse = 1, Medium = 2, Dense = 3
Shape of the tips of sterile sepals	SSS	Pointed = 1, Round = 2
Degree of vesiculation in sterile sepals	DCSS	None or weak = 1, moderate = 2
Cross-sectional Shape of Sterile Sepals	CSSS	Flat = 1, Slightly Concave = 2
The margins of the sterile sepals are curved inward	SSIC	None or very weak = 1, weak = 2
Folds on the sterile sepals	SSWF	None or very weak = 1, moderate = 2, strong = 3
Notched margins on the sterile sepals	SSNM	None = 1, Some = 2, All = 3
Main Color of Sterile Sepals	SCSP	White = 1, Green = 2, Pink = 3, Yellow = 4, Blue = 5, Purple = 6
Number of rounds of sterile sepals	NWSC	Single round = 1, two rounds = 2, multiple rounds = 3
Overlap of Sterile Sepals	ODSS	Weak = 1, Medium = 2, Strong = 3
Plant height	PH	During peak flowering, measure the vertical height from the base to the top of the plant (m); for each accession, measure three plants and calculate the average.
Logarithm of the number of lateral veins	NLV	Count the number of lateral veins on either side of the midrib of mature leaves; measure 10 leaves per accession and calculate the average.
Petiole Length	PL	Measure with a ruler (in cm); measure 10 leaves from each accession and calculate the average.
Leaf Length	LGL	Measure with a ruler (in cm); measure 10 leaves from each accession and calculate the average.
Leaf width	LB	Measure with a ruler (in cm); measure 10 leaves from each accession and calculate the average.
Number of sepals per flower	NSPF	Count the number of sterile sepals per flower; measure 30 flowers per germplasm sample and calculate the average.
Diameter of a single sepal	DSS	Measure using a vernier caliper (cm); measure 30 flowers or 30 sepals from each accession and calculate the average.
Length of Sterile Sepals	SSE	Measure using a vernier caliper (cm); measure 30 flowers or 30 sepals from each accession and calculate the average.
Width of sterile sepals	SSB	Measure using a vernier caliper (cm); measure 30 flowers or 30 sepals from each accession and calculate the average.

## Data Availability

The original contributions presented in the study are included in the article. Further inquiries can be directed to the corresponding author.

## Supplementary Materials

The following supporting information can be downloaded at: https://www.mdpi.com/article/10.3390/plants15182821/s1.
